# Interaction of phosphorus and water supply regulates the maize root system and phosphorus-use efficiency

**DOI:** 10.3389/fpls.2025.1665508

**Published:** 2026-01-27

**Authors:** Maoying Wang, Jie Xu, Yang Lyu, Mengjie Luo, Yucui Sun, Shengjia Ye, Lingyun Cheng, Zed Rengel, Jianbo Shen

**Affiliations:** 1 State Key Laboratory of Nutrient Use and Management, College of Resources and Environmental Sciences, National Academy of Agriculture Green Development, Key Laboratory of Plant-Soil Interactions, Ministry of Education (MOE), China Agricultural University, Beijing, China; 2 Research and Development Centre, Yunnan Yuntianhua Co., Ltd., Kunming, China; 3 Soil Science and Plant Nutrition, The University of Western Australia (UWA) School of Agriculture and Environment, The University of Western Australia, Perth, WA, Australia; 4 Institute for Adriatic Crops and Karst Reclamation, Split, Croatia

**Keywords:** maize, phosphorus fertilizers, water supply, rhizosphere processes, P-use efficiency, interactions

## Abstract

**Introduction:**

Improving phosphorus (P)-use efficiency (PUE) while increasing crop yield is one of the greatest challenges in sustainable P management for sustainable agriculture. Types of P fertilizers and soil water supply impact P availability and crop growth, but how to optimize P fertilizer and water supply to enhance the foraging capacity of roots for P remains unclear. This study was aimed at characterizing the effects of different combinations of P fertilizers and water supply on maize growth, root properties and PUE in calcareous soil.

**Methods:**

A pot experiment with four P fertilizers [monoammonium phosphate (MAP), diammonium phosphate (DAP), ammonium polyphosphate (APP) and urea phosphate (UP)] was conducted under well-watered (watered) and water-deficit (dry) conditions using maize (*Zea mays* L.) in a greenhouse during the seedling stage.

**Results:**

The interaction between P fertilizers and water supply significantly promoted the growth and P uptake of maize by modifying the root morphological and physiological traits. MAP and APP exhibited greater (by up to 62%) total root length in the watered than the dry treatments, resulting in a significant increase in the efficiency of root P acquisition. The APase activity in the rhizosphere soil of MAP and DAP declined (by 37%-62%) significantly, and the rhizosphere soil pH in the DAP treatment was 0.4 units lower in the watered than the dry treatments. APP improved the soil P availability more than the other P fertilizers (17%-41% higher in soil Olsen-P concentration) regardless of water supply.

**Conclusion:**

Optimal combination of P fertilizers and water supply promotes maize growth and PUE due to stimulating the root capacity to forage for nutrient and water resources by regulating the root morphological and physiological traits. Engineering root/rhizosphere by manipulating the interactions of P fertilizer types and water supply can improve nutrient use-efficiency and sustainable crop production.

## 1 Introduction

Phosphorus (P) is an essential macronutrient for plant growth, involved in the composition of important structures, cell energy transfer, and various metabolic processes (Al-Juthery et al., 2021; Hu et al., 2023). The application of P fertilizers is important in ensuring food security (Langhans et al., 2022). The yield of three major grain crops (rice, wheat and maize) increased by 16% in China in response to mineral P fertilizer applications (Gong et al., 2022). However, excessive P fertilization occurs regularly (Sattari et al., 2014; Li et al., 2016). The use efficiency of large amounts of P fertilizer applied is generally less than 20% in the first cropping season due to the adsorption/fixation of P by soil (e.g., Simpson et al., 2011; Zhang et al., 2019), which has resulted in the accumulation of P in soils and even leaching/runoff of P to waters, leading to severe environmental problems, such as water eutrophication (Zhu et al., 2018; Yan et al., 2021). Furthermore, phosphate rock (mined from the ground and used in the production of P fertilizers) is a non-renewable resource made by natural processes over geological timeframes. Despite some new discoveries of phosphate rock deposits (e.g., in Norway in 2018) that are quite deep and therefore technically challenging and expensive to exploit, the inescapable inference is that the humanity will run out of phosphate rock to mine for producing P fertilizers over the course of the next few generations (cf. Geissler et al., 2015). Therefore, it is necessary to improve P-use efficiency (PUE) while increasing crop yield for achieving sustainable P management and developing green agriculture.

Soil water significantly affects P availability because (i) the movement of P is mainly through diffusion (with a diffusional pathway lengthening significantly due to tortuosity under low soil water contents) and (ii) the P biogeochemical cycles, including sorption/desorption and precipitation/dissolution, are highly sensitive to soil water content (Plett et al., 2020; Maharajan et al., 2021). Hence, low soil water content may significantly reduce P accumulation by plants and grain yield (e.g. by 40% and 60%, respectively, in wheat; He et al., 2017).

Climate change resulting from global warming is increasing precipitation in some, but exacerbating aridity in other, parts of the world. Aridity leads to water stress, reducing crop yield and agricultural efficiency (Shayanmehr et al., 2022; Kim et al., 2023). However, under water stress conditions, maize root growth was stimulated [the root/shoot ratio and specific root length increased, and the inorganic phosphate transporter genes were up-regulated (Zhan et al., 2019)]. Plants allocate more biomass to roots to improve the soil exploration and increase P uptake under P-deficiency conditions (Lambers et al., 2006). Within a certain range of reduced soil water content, P-uptake efficiency and total P uptake would increase due to the root system alterations. However, decreased P diffusion in partially dry soil would counteract the beneficial effects of the improved root system on total P uptake (Bauw et al., 2019).

Different P fertilizers vary in their impact on crop growth and nutrient-use efficiency because of their specific physical and chemical properties. These properties, which control the immediate reaction of P fertilizers with soil surface or solution, primarily include the phosphate ion concentration and its speciation, the form and concentration of the accompanying cation and the pH of the solution. The reactions of the different P fertilizers and their fertilizer solutions with soils can be categorized as follows: (1) triple superphosphate (TSP) and single superphosphate (SSP); (2) monoammonium phosphate (MAP) and diammonium phosphate (DAP); (3) ammonium polyphosphates (APP) (Hedley and Mclaughlin, 2005). Based on these specific properties, the fertilizer efficiency and agronomic effectiveness of P fertilizers also vary significantly across different soil environments. For example, the application of acid or neutral fertilizers, including MAP, APP, and urea phosphate (UP), resulted in higher maize yield and economic benefits in the calcareous soil compared with alkaline fertilizers (e.g. DAP) (Wang et al., 2024b; Tian et al., 2024). By contrast, in acid soil, the maize P-uptake efficiency and partial factor productivity of calcium magnesium phosphate (CMP) and DAP were higher than those of SSP, MAP and APP (Zhao et al., 2021). APP reduced the fixation/sorption and increased the desorption/dissolution of P due to the simultaneous presence of soluble orthophosphate, slowly hydrolyzed pyrophosphate and more condensed P forms (Yuan et al., 2022).

Currently, the two high-grade P fertilizers (MAP and DAP) are predominantly used in agricultural production in China because of their perceived yield-increasing effect, even though these fertilizers are associated with increasing soil residual P and exacerbated P losses (Gong et al., 2022). The production of DAP and MAP has accounted for about 80% of the total P fertilizer production in China since 2014 (Zhang et al., 2017). Low-grade P fertilizers such as SSP and CMP are gradually declining on the market because their low nutrient content is not favored by farmers. New fertilizers (such as APP, UP, and other slow-release P fertilizers) have not been widely accepted due to their high prices, which significantly limited the transformation and upgrade of P fertilizer industry as well as the development of green agriculture. The research on the use efficiency of different P fertilizers in different regions or environmental conditions is urgently needed to provide scientific support for the efficient utilization of existing P fertilizers and the formulation of new P fertilizers. Current research is mainly focused on P application rates under various crop-producing conditions, but the studies comparing different P fertilizers for PUE via influencing root growth and rhizosphere processes are relatively rare, and this paucity is even more obvious regarding the interaction of P fertilizer types and water supply.

This study was aimed at investigating the effects of two widely used P fertilizers (MAP, DAP) and two new P fertilizers (APP, UP) on the root and rhizosphere processes of maize under two contrasting water supplies to test the following hypotheses: (1) different P fertilizers would differentially influence maize growth and P uptake by altering root morphology and rhizosphere processes, and this effect would be modified by soil water supply; and (2) optimizing the interaction between P fertilizer type and soil water content can increase PUE and maize growth, depending on the properties of P fertilizers.

## 2 Materials and methods

### 2.1 Fertilizer properties

The pH of fertilizer solution was measured by a pH meter (Mettler Toledo, FE20) at a fertilizer-water ratio of 1:5 according to GB/T 18877-2020 (National Standardization Administration, 2020). The Quin molybdenum ketone gravimetric method was used to determine the available P content in P fertilizers according to GB/T 8573-2017 (National Standardization Administration of China, 2017). The distillation–titration method was used to determine the total nitrogen content in P fertilizers according to GB/T 8572-2010 (National Standardization Administration of China, 2010).

One g of P fertilizer was placed in 10 cm Petri dish followed by adding 10 mL of deionized water (temperature: 21.5°C). An infrared camera (FLIR T660, FLIR Systems, Inc., Wilsonville, OR, USA) was used to record the images of temperature changes over time (0–20 min) in different P fertilizer aqueous solutions. The FLIR Tools 5.0 (FLIR Systems, Inc., Wilsonville, OR, USA) was used to analyze the images to quantify temperature dynamics in different treatments.

### 2.2 Experimental set-up

The pot experiment was conducted in a greenhouse at the China Agricultural University, Beijing (40°1′46″N, 116°17′11″E). The experiment tested two factors. The first factor (fertilizer type) comprised two P fertilizers widely used by farmers (MAP, DAP) and two new P fertilizers (APP, UP) (for their properties see **Table 1**). In addition, no P fertilizer (CK) was used as a control. The second factor was water treatment with well-watered (watered) and water-deficit (dry), with daily watering to 70% (watered) and 40% (dry) of field capacity (i.e. soil water content is 25%). Water treatments commenced 18 days after sowing (prior to that, all pots were watered to 70% of field capacity to maintain the seedling growth). There were 10 treatments combinations with four replicates per treatment arranged in a randomized complete block design.

**Table 1:** The characteristics of P fertilizers tested in the study.

P fertilizers	pH	N concentration (g kg^-1^)	N forms	P concentration (g kg^-1^)	P forms
Monoammonium phosphate	4.46	120	NH_4_^+^	270	H_2_PO_4_^-^
Diammonium phosphate	8.29	210	NH_4_^+^	230	HPO_4_^2-^
Ammonium polyphosphate	6.39	150	NH_4_^+^	250	P_n_O_3n+1_^(n+2)-^
Urea phosphate	1.60	177	-CO-NH_2_	196	H_2_PO_4_^-^; HPO_4_^2-^

### 2.3 Soil and plant management

Calcareous silt loam soil that has not been fertilized for a long time was collected from the Shangzhuang Experimental Station of China Agricultural University in Beijing (40°8′5″N, 116°11′3″E). Soil was air-dried, sieved (<2 mm) and analyzed. Soil characteristics were: pH 8.2 (1:5, soil:water), total N 0.72 g kg^-1^, Olsen-P 2.6 mg kg^−1^, exchangeable K 32.3 mg kg^−1^, and organic carbon 11.7 g kg^-1^. The basal nutrients as solutions were uniformly added to soil before treatments as follows (mg kg^−1^ soil): 200 K (K_2_SO_4_), 45 Ca (CaCl_2_), 4.22 Mg (MgSO_4_·7H_2_O), 0.88 Fe (EDTA-FeNa), 2.17 Mn (MnSO_4_·H_2_O), 2.26 Zn (ZnSO_4_·7H_2_O), 0.51 Cu (CuSO_4_·5H_2_O), 0.12 B (H_3_BO_3_) and 0.14 Mo [(NH_4_)_6_Mo_7_O_24_·4H_2_O].

Different P fertilizers were mixed with the soil at 150 mg P kg^−1^. In addition, due to the different amounts of N in the treatment fertilizers, the specific amounts of N were supplemented in the form of urea to various pots so that each pot received 200 mg N kg^−1^ soil. The final formulations of MAP, DAP, and APP all contain a blend of ammonium nitrogen and amide nitrogen, while UP contains only amide nitrogen. The ratios of ammonium nitrogen to amide nitrogen in each treatment are as follows: 3:7 for MAP, 7:3 for DAP, 5:5 for APP, and 0:10 for UP.

Similarly-sized seeds of the hybrid maize variety Zhengdan 958 (*Zea mays* L. cv. ZD958) were surface-sterilized by 10% v/v H_2_O_2_ for 30 min, rinsed with deionized water, soaked for 12 h in saturated CaSO_4_ solution, and then germinated in Petri dishes covered with filter paper moistened with deionized water in darkness at 25°C. Four uniformly germinated seeds were sown per pot containing 1.5 kg of air-dried soil and were thinned to two plants after 8 days.

### 2.4 Sampling and measurements

The plants were harvested by cutting shoots just above the soil surface after 46 days. Roots were carefully separated from soil and shaken to remove bulk soil.

Roots with rhizosphere soil attached were dunked into appropriate amounts of 0.2 m*M* CaCl_2_ solution and gently shaken until as much rhizosphere soil as possible was dislodged. Roots were removed, and the suspension was gently shaken (Pearse et al., 2007). Two 0.5 mL aliquots of soil suspension were transferred into 2 mL centrifuge tubes to measure acid phosphatase (APase) activity, and another 8 mL was collected for measuring rhizosphere pH. The APase activity in the rhizosphere soil was measured by the colorimetric method using an enzyme-linked immunosorbent assay (ELISA) reader. Specifically, 0.4 mL of sodium acetate buffer (pH 5.2) and 0.1 mL of 0.15 *M p*-nitrophenyl phosphate were added to 2 mL centrifuge tubes containing 0.5 mL of soil suspension followed by incubation at 30°C for 30 min. Afterwards, 0.5 mL of 0.5 *M* sodium hydroxide was added to terminate the reaction before colorimetric determination at 405 nm (Alvey et al., 2001). The pH of the rhizosphere soil suspension was determined using a pH meter (Mettler Toledo, FE20). To eliminate the influence of different soil-solution ratios on the measurement results caused by the difference in the weight of rhizosphere soil collected from different samples, the unified soil-solution ratio (1:2.5, the solution refers to 0.2 m*M* CaCl_2_ solution) was corrected by an equation according to Li et al. (2010).

All visible roots were collected and rinsed under running water. The roots were scanned using an EPSON scanner at 400 dpi (Epson Expression 1600 pro, Model EU-35, Japan). Root images were analyzed using WinRhizo software (Regent Instruments Inc., Quebec, QC, Canada) to calculate root length and average diameter. Afterwards, roots were oven-dried at 75°C for 48 h before weighing.

Bulk soil samples were air-dried, sieved (2 mm), and stored for analysis. Olsen-P was extracted using 0.5 *M* NaHCO_3_ (2.5 g soil, 50 mL solution, 25°C, shaken for 30 min), followed by the colorimetric measurement of inorganic P using the molybdate-ascorbic acid method (Murphy and Riley, 1962).

Shoots were oven-dried at 105°C for 30 min and then at 75°C for 48 h before weighing. Dried shoots were ground into powder and digested with 5 mL of concentrated H_2_SO_4_ and 2 mL of 30% v/v H_2_O_2_. Shoot P concentration was determined by the molybdo-vanadophosphate colorimetric method (Johnson and Ulrich, 1959). The following formula was used to calculate PUE:


PUE(%)=(shoot P content of fertilization treatment–shoot P content of CK)/total P supplied by fertilizer.

### 2.5 Statistical analysis

Two-way analysis of variance (ANOVA) considering P fertilizer types × water treatments were performed to assess the treatment difference in soil Olsen-P concentration, maize growth, root traits and P uptake using SPSS 22.0 (IBM SPSS Inc., Chicago, IL, USA). Significant differences among means were separated by the Tukey test (HSD) at 5%, 1% and 0.1% probability level (0.01< *p* ≤ 0.05, 0.001< *p* ≤ 0.01, and *p* ≤ 0.001).

## 3 Results

### 3.1 Soil Olsen-P concentration

**Figure 1:**
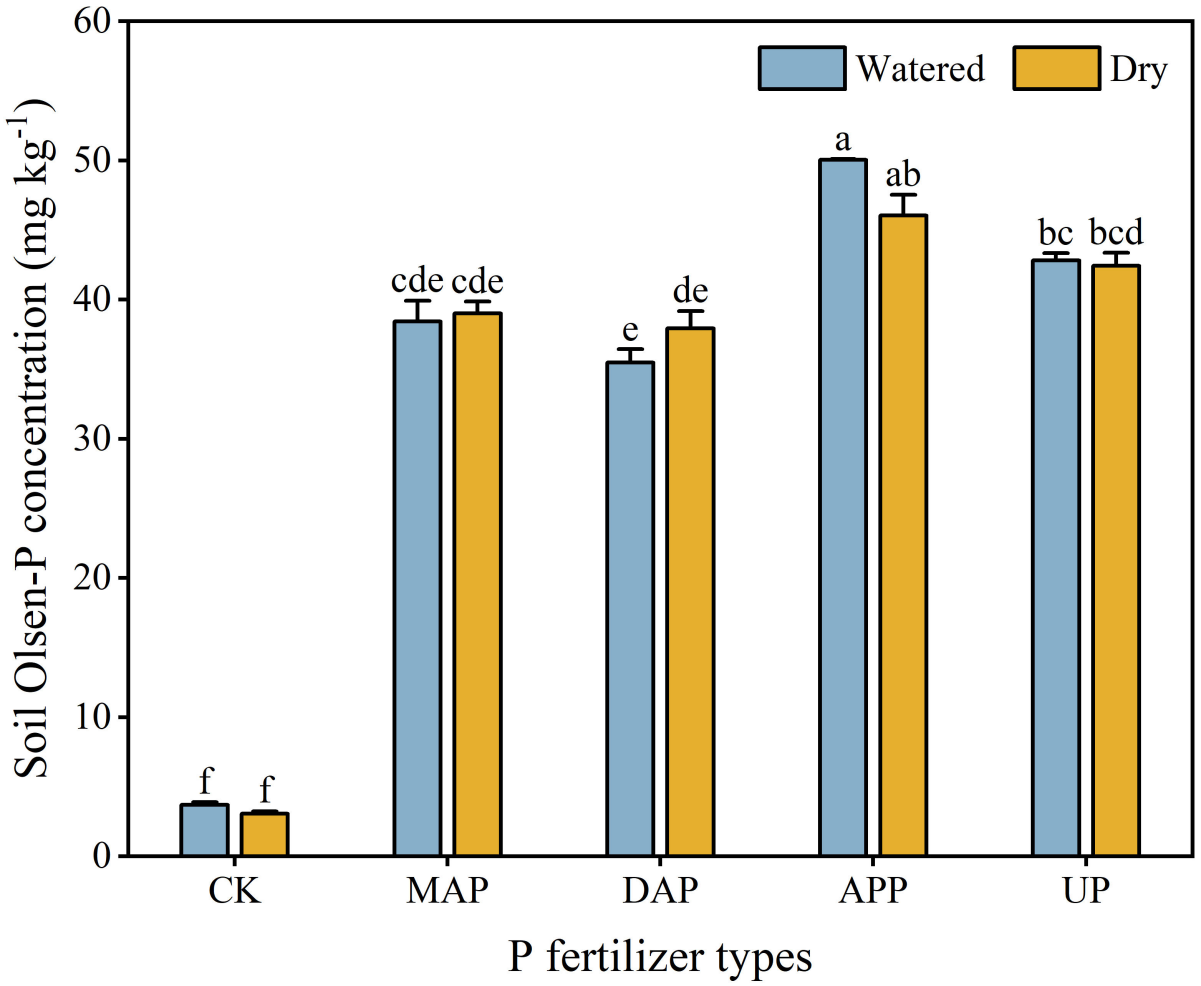
Effects of the interaction between P fertilizers and water supply on soil Olsen-P concentration. CK, no P fertilizer; MAP, monoammonium phosphate; DAP, diammonium phosphate; APP, ammonium polyphosphate; UP, urea phosphate. Each value is the mean (+ se) of four replicates. Soil Olsen-P concentration was significantly affected by the interaction of P fertilizer × water supply; therefore, different lowercase letters show statistically significant differences among all the treatments (*P* ≤ 0.05).

Soil Olsen-P concentration was significantly affected by the interaction between P fertilizers and water supply (**Table 2**). In the watered treatment, APP had the highest soil Olsen-P concentration (17%-41% higher than other P fertilizers). There was no difference in soil Olsen-P concentration between watered and dry treatments for the given P fertilizer (**Figure 1**).

**Table 2:** The effects of P fertilizer, water supply and their interaction on soil Olsen-P concentration and maize growth.

Parameter	*P*-values		
	P fertilizer	Water supply	Fertilizer × Water
Soil Olsen-P concentration	**<0.001**	0.502	**0.029**
Shoot biomass	**<0.001**	**<0.001**	**<0.001**
Root biomass	**<0.001**	**<0.001**	**0.003**
Root/shoot ratio	**<0.001**	**<0.001**	**<0.001**
Total root length	**<0.001**	**<0.001**	**<0.001**
Average root diameter	**<0.001**	**<0.001**	**<0.001**
Rhizosphere soil pH	**<0.001**	0.424	**<0.001**
APase activity in rhizosphere soil	**<0.001**	**<0.001**	**<0.001**
Shoot P concentration	**<0.001**	**<0.001**	0.153
Shoot P content	**<0.001**	**<0.001**	**<0.001**
PUE	**<0.001**	**<0.001**	**<0.001**

Significant P values (*P* ≤ 0.05) are in bold.

### 3.2 Shoot and root biomass of maize

**Figure 2:**
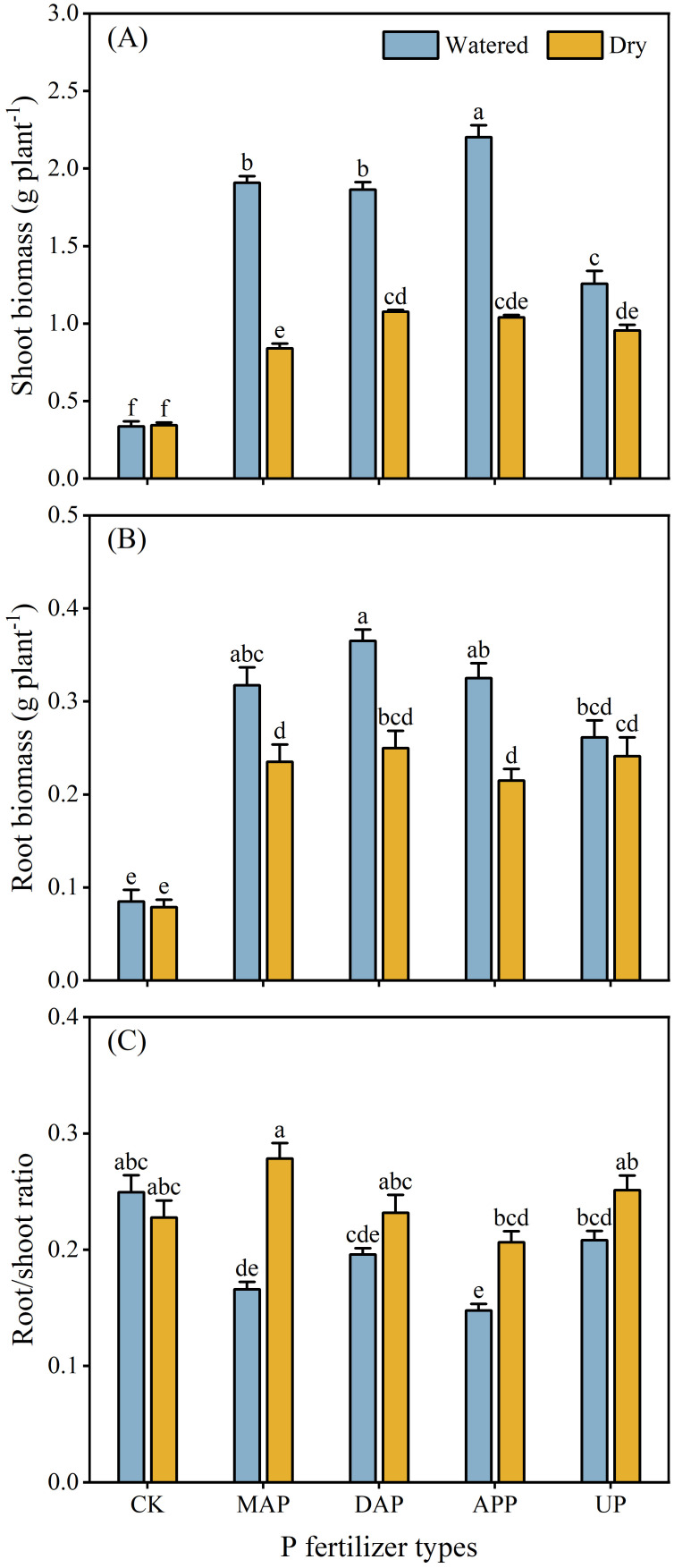
Effects of the interaction between P fertilizers and water supply on shoot biomass **(A)**, root biomass **(B)** and root/shoot ratio **(C)** of maize. For explanation of acronyms, see **Figure 1**. Each value is the mean (+ se) of four replicates. Shoot biomass, root biomass and root/shoot ratio were significantly affected by the interaction of P fertilizer × water supply; therefore, different lowercase letters show statistically significant differences among all the treatments (*P* ≤ 0.05).

The interaction between P fertilizers and water supply significantly influenced biomass of maize shoots and roots and root/shoot ratio (**Table 2**). No difference was noted between watered and dry treatments in shoot biomass in CK, whereas the shoot biomass was greater in watered than dry treatments in all fertilizer treatments (31% greater in UP and 73%-127% greater in case of MAP, DAP and APP) (**Figure 2A**). The root biomass of MAP, DAP and APP significantly increased (by 35%-51%) in the watered treatment compared with the dry treatment, but no differences occurred in the treatments with UP and CK (**Figure 2B**). Consequently, the root/shoot ratio of P fertilizer treatments was lower in watered than dry treatments (40% lower in MAP and 28% lower in APP, but no differences were recorded in the treatments with DAP and UP) (**Figure 2C**).

### 3.3 Root morphology

**Figure 3:**
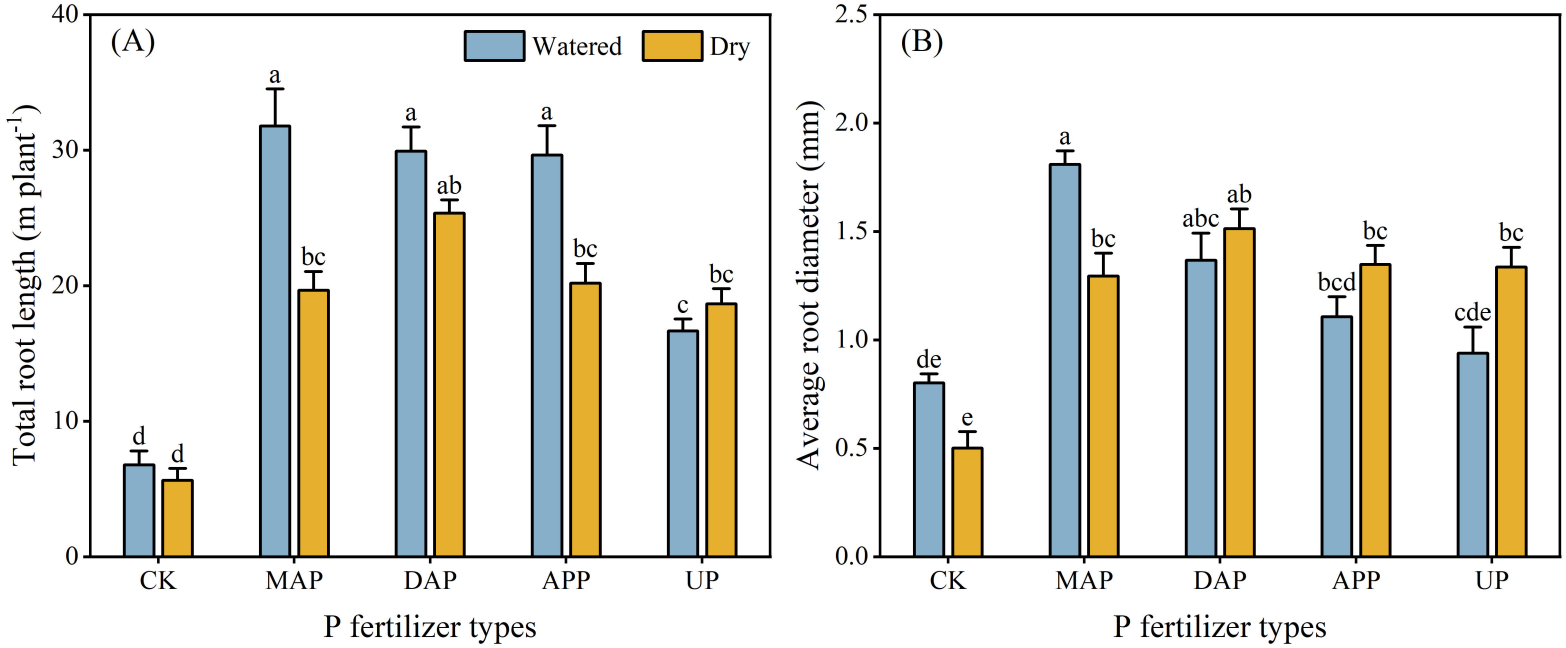
Effects of the interaction between P fertilizers and water supply on total root length **(A)** and average root diameter **(B)**. For explanation of acronyms, see **Figure 1**. Each value is the mean (+ se) of four replicates. Total root length and average root diameter were significantly affected by the interaction of P fertilizer × water supply; therefore, different lowercase letters show statistically significant differences among all the treatments (*P* ≤ 0.05).

The total root length and average root diameter were significantly affected by the interaction between P fertilizers and water supply (**Table 2**). Compared with dry treatments, MAP and APP significantly increased the total root length (by 47%-62%) in watered treatments. No differences were found between the watered and the dry treatments in case of CK, DAP and UP (**Figure 3A**). The average root diameter was greater (by 40%) in watered than dry treatment only in case of MAP, with a non-significant trend of the opposite for the DAP, APP and UP treatments (**Figure 3B**).

### 3.4 Root physiological traits

**Figure 4:**
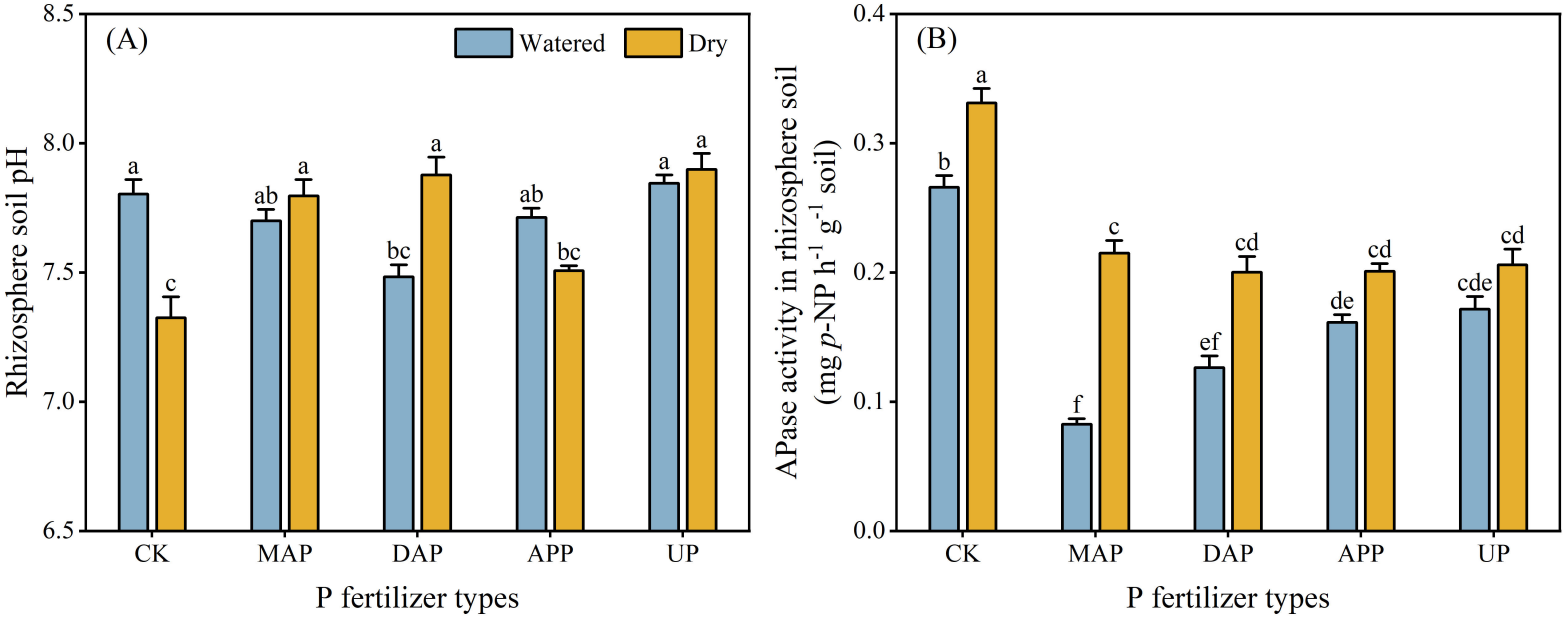
Effects of the interaction between P fertilizers and water supply on pH **(A)** and APase activity **(B)** in rhizosphere soil. For explanation of acronyms, see **Figure 1**. Each value is the mean (+ se) of four replicates. The pH and APase activity in rhizosphere soil were significantly affected by the interaction of P fertilizer × water supply; therefore, different lowercase letters show statistically significant differences among all the treatments (*P* ≤ 0.05).

The interaction between P fertilizers and water supply significantly influenced rhizosphere soil pH and APase activity in rhizosphere soil (**Table 2**). Compared with dry treatments, the rhizosphere soil pH was significantly decreased in DAP (by 0.4 units) and increased in the CK (by 0.5 units) in the watered treatments (**Figure 4A**). There was no significant difference between watered and dry treatments in the rhizosphere soil pH of MAP, APP and UP. The APase activity in rhizosphere soil of CK, MAP and DAP significantly increased (by 25%, 160% and 58%, respectively) in dry compared with watered treatments, whereas no difference between the watering treatments was noted for APP and UP (**Figure 4B**).

### 3.5 P uptake and use efficiency

**Figure 5:**
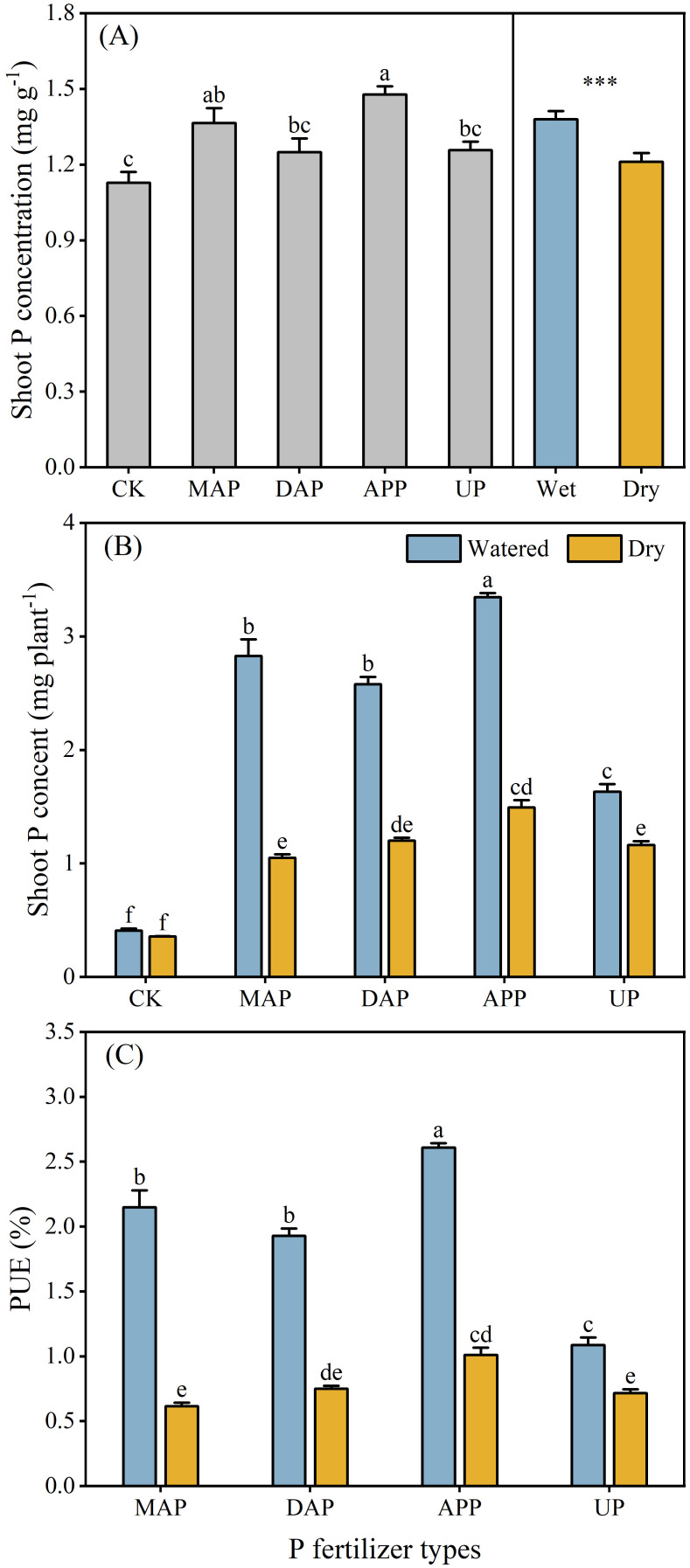
Effects of different P fertilizers and water supplies on shoot P concentration **(A)**, shoot P content **(B)** and PUE **(C)**. For explanation of acronyms, see **Figure 1**. When the interaction was not significant [in **(A)**], only the significant main effects were presented. Different lowercase letters show statistically significant differences among different P fertilizers (*P* ≤ 0.05), and the t-test was run to assess the difference between the watered and dry treatments. ***P < 0.001. The interaction between P fertilizer and water supply was significant in **(B)** and **(C)**; therefore, different lowercase letters show statistically significant differences among all the treatments. Each value is the mean (+ se) [in **(A)**, n = 8 for P fertilizers and n = 20 for water supplies; in **(B, C)**, n = 4].

The P concentration of maize shoots was affected by P fertilizer types and water supply, but not by their interaction (**Table 2**). The shoot P concentration in the APP treatment was significantly higher (by 17%-31%) than in the CK, DAP and UP treatments, and not different from MAP (**Figure 5A**). The watered treatment significantly increased the shoot P concentration of maize (by 14%) compared with dry treatment.

The interaction between P fertilizers and water supply significantly influenced shoot P content and PUE (**Table 2**). No difference in shoot P content was noted between watered and dry treatments in CK, whereas the shoot P content was significantly greater in watered than dry treatments for all fertilizers (40% in case of UP and 115%-169% greater for MAP, DAP and APP) (**Figure 5B**). Similarly, PUE was significantly higher (52% for UP and 157%-249% in case of MAP, DAP and APP) in watered than dry treatments (**Figure 5C**).

## 4 Discussion

Exploring the effects of different P fertilizers on the root growth and P uptake of maize in dependence of soil water content provides important evidence for achieving the precise and efficient use of P fertilizers to underpin sustainable P management. The present study indicated that the root morphological and physiological traits of maize exhibited differences in response to the interaction between P fertilizers and water supply, which were associated with improved nutrient uptake and stimulated maize growth.

APP maintained the highest soil Olsen-P concentration (especially in watered treatments) compared with other fertilizers (**Figure 1**). Given that APP contains orthophosphate (ortho-P, H_2_PO_4_^-^, HPO_4_^2-^, PO_4_^3-^) and polyphosphates (poly-p, P_n_O_3n+1_^(n+2)-^, n ≥ 2), it can quickly provide ortho-P in the early stage of maize growth, and continuously provide available P from poly-P in the later stage. The poly-P can slowly hydrolyze into ortho-P that can be directly absorbed by plants (McBeath et al., 2007; Zhou et al., 2023). Moreover, the poly-P can also decrease P fixation and increase soil available P by preventing Ca-P formation or retarding the ripening of Ca-P to more stable mineral in calcareous soils (Gao et al., 2020; Yuan et al., 2022). Hence, poly-P can improve P availability in soil and P uptake by crops compared with traditional ortho-P fertilizers (Weeks and Hettiarachchi, 2019; Erel et al., 2023), such as MAP and DAP, because of ready fixation of ortho-P by soil (Rajput et al., 2014).

In calcareous soils, the phosphate from UP takes time to transform into the P forms that can be absorbed by plants, leading to insufficient supply of P in the early stage of maize growth (Ji et al., 2021). A temperature decrease caused by UP dissolution (**Supplementary Figure S1**) (Long et al., 2022) might further prolong the transformation time and also slow the germination of seeds compared with other P fertilizers if the fertilizer is close to seeds (Sbrussi and Zucareli, 2014). Based on the above results, it is recommended that the combined application of readily-available P and slow-release P like APP should be further tested to effectively reduce P fixation and improve PUE.

The major root morphology traits (e.g., total root length in MAP and APP, root diameter in MAP) or physiological traits (rhizosphere soil pH in DAP, APase acitivity in rhizosphere soil in MAP and DAP) of maize treated with MAP, DAP and APP differed in the two contrasting soil water conditions, but not in case of UP (**Figures 3**, **4**). This may be due to the presence of NH_4_^+^-N in MAP, DAP and APP. Crops release protons to the rhizosphere in the process of absorbing NH_4_^+^-N, which not only acidified the rhizosphere, but also accelerated the cell divisions and cell wall elongation of root meristematic cells, promoting root proliferation (Bloom et al., 2002; Lin et al., 2021). Longer roots mean increased root-soil contact area, thus improving root uptake of available nutrients from soils (Jing et al., 2022). Although urea (the N form in UP) can also hydrolyze to produce NH_4_^+^-N, thereby influencing root growth, its complete hydrolysis to release NH_4_^+^-N requires up to four days even in fertile soil with 18% soil water content (Skiba and Wainwright, 1984). Moreover, the high pH of calcareous soils further restricts this conversion. Root proliferation was greater with a combined supply of P and NH_4_^+^-N than with other N forms, such as nitrate or urea (Jing et al., 2012), which aligns with the findings in this study. Specifically, MAP and APP showed a significant increase in total root length of maize in the watered compared with the dry treatments (**Figure 3**). Maize allocated relatively more photosynthates to shoots under the conditions favoring efficient uptake of nutrient by roots (Poorter and Nagel, 2000; Nielsen et al., 2001; Zhang et al., 2023), resulting in an increase in shoot biomass and decreased root/shoot ratio in the treatments with MAP and APP (**Figure 2**). The application of DAP significantly reduced the rhizosphere soil pH in the watered compared with the dry treatment (**Figure 4A**), likely due to its high NH_4_^+^-N content under optimal soil water content, whereby soil acidification may enhance the P availability in the rhizosphere soil, thus increasing the P uptake by maize (**Figure 5**). However, there was no difference in the root/shoot ratio of DAP in the two watering treatments, even though the root and shoot biomass significantly increased in the watered treatment (**Figure 2**). It indicated that the nutrient uptake efficiency of roots was lower in the DAP than the MAP and APP treatments, although DAP had the highest proportion of NH_4_^+^-N. An excessively high level of NH_4_^+^-N can cause ammonium toxicity, which is detrimental to plant and root growth (Hachiya et al., 2021; Zhang et al., 2021). Another possible reason for this is that the high pH of DAP solution in the early stage of dissolution weakens the positive effect of rhizosphere acidification on root growth and increases the formation of insoluble Ca phosphates in calcareous soil (Hedley and Mclaughlin, 2005). By contrast, the acidic fertilizers exhibited higher root and fertilizer efficiency in calcareous soil (Wang et al., 2024b; Tian et al., 2024), which was consistent with the results of the present study. However, compared with other P fertilizers, the maize shoot and root biomass and PUE of UP showed the smallest increase in the watered vs. dry treatments despite the UP solution having the lowest pH of all fertilizers tested (**Figures 2**, **5**). This further demonstrates that the fertilizer efficacy is subject to the collective influence of factors such as nutrient types, forms, and pH (Hedley and Mclaughlin, 2005). And the results might suggest that the maize root growth was more effective than the fertilizer pH in regulating the responses to environmental and nutrient changes, emphasizing the importance of engineering the rhizosphere microenvironment and the soil macro-environment to promote nutrient-use efficiency and crop growth as opposed to relying solely on high fertilizer input (Wang et al., 2020; Wang et al., 2024a). In summary, among the four P fertilizers evaluated, APP demonstrates higher agronomic performance. However, due to high raw material costs and substantial energy consumption, APP products are priced 1.7 to 2.1 times higher than MAP and DAP, which limits their widespread adoption (Lin and Yin, 2014; Kang and Chu, 2018). A key challenge is to achieve low-cost, continuous and large-scale production of APP. In recent years, some progress has been made in reducing APP production costs through the use of low-cost feedstocks such as wet-process phosphoric acid and its by-product raffinate, alongside process optimizations that reduce material requirements. The resulting low-cost and high-quality products have established a foundation for large-scale production and widespread promotion of APP (Li, 2025).

The shoot biomass (**Figure 2**) and PUE of maize (**Figure 5**) treated with four P fertilizers were significantly higher in the watered than the dry treatments, suggesting that the nutrient uptake by roots decreased in the dry treatment, limiting maize growth. One reason is that low soil water content can reduce the P dissolution and diffusion in soil, leading to a decrease in P availability (Plett et al., 2020; Maharajan et al., 2021). Moreover, aridity also directly affects functioning of the root system, reducing hydraulic conductivity and stomatal conductance and restricting root-to-shoot nutrient transport (Lefebvre et al., 2011). The root physiological response to P stress (higher APase activity in the rhizosphere soil in the CK than in any fertilizer treatment) and water shortage in the MAP and DAP treatments (higher APase activity in the rhizosphere soil in the dry compared with the watered treatments) would have likely resulted in increased P availability from organic P sources (**Figure 4B**). Similar results were also found in the previous studies (Xia et al., 2020; Jing et al., 2022). By contrast, the response to P stress also included an increase in exudation of protons and strong acidification of the rhizosphere soil in the previous studies (Liu et al., 2004; Shen et al., 2011), but in the present study the rhizosphere soil acidification was similar in the CK and most fertilizer treatments (except DAP) in the watered conditions, but this acidification was greater in the CK compared with the MAP, DAP and UP treatments under water shortage (**Figure 4A**). A possible reason for these findings might have been the interactive effects between P fertilizer and water supply. Exacerbated aridity can increase soil pH, which would be associated with depressed nutrient mobility and availability (Suriyagoda et al., 2014; Zhan et al., 2019) as well as decreased proton release accompanying low cation uptake rates (Bloom et al., 2002; Yan et al., 2002). In conclusion, the root growth and rhizosphere processes can effectively be regulated by using specific P fertilizers to suit specific soil conditions (such as low soil water content), thus affecting crop growth and uptake of fertilizer nutrients. Precise application of P fertilizers and developing new P-based smart fertilizers should be implemented to enhance sustainability of agricultural production based on better understanding of the interactive soil-crop-environment system to achieve the high crop yields with efficient use of P fertilizers and small environmental footprint.

## 5 Conclusion

In the present study, MAP, DAP and APP significantly promoted maize shoot growth and PUE in the watered treatments by modifying the root morphology and physiological traits. The best agronomic performance was observed with APP, followed by that of MAP. In addition, APP maintained a high soil Olsen-P concentration in both watered and dry treatments. The responses of maize root growth and P availability differed depending on the combinations of P fertilizer and water supply, emphasizing the necessity to coordinate the choice of P fertilizer with the interactive crop-soil-environment systems to improve both nutrient uptake by roots and the fertilizer use efficiency for sustainable crop production.

## Data Availability

The raw data supporting the conclusions of this article will be made available by the authors, without undue reservation.
