# Supplementary material for: Interaction of phosphorus and water supply regulates the maize root system and phosphorus-use efficiency
Source: Front Plant Sci. 2026 Jan 27;16:1665508. doi: 10.3389/fpls.2025.1665508 (PMC12886405; doi:10.3389/fpls.2025.1665508)
Supplement: Supplementary file 1 [file DataSheet1.docx]

***Supplementary Material***

**Figure S1**


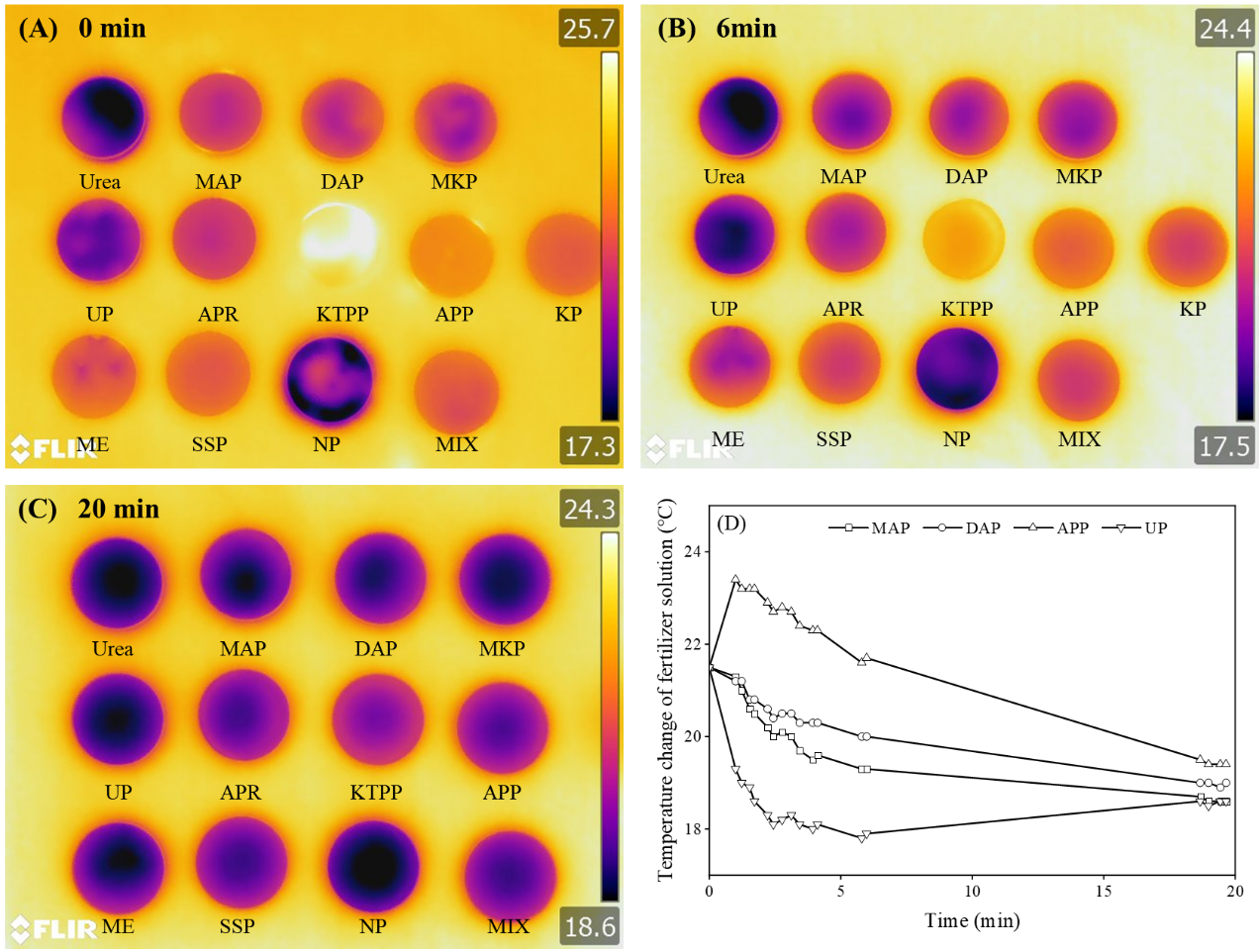


**Figure S1.** The infrared thermography images in of different P fertilizer solutions: at 0 min **(A)**, 6 min **(B)**, and 20 min **(C)**. The time-course of temperature changes in aqueous solutions of different P fertilizers **(D)**. MAP, monoammonium phosphate; DAP, diammonium phosphate; MKP, monopotassium phosphate; UP, urea phosphate; APR, ammonium pyrophosphate; KTPP, potassium tripolyphosphate; APP, ammonium polyphosphate; KP, potassium phosphite; ME, the MicroEssentials product by Mosaic fertilizer, LLC; SSP, single superphosphate; NP, ammonium nitrate phosphate; MIX, the 1:1 blend of SSP and APR by P content.
